# International workshop: what is needed to ensure outcome measures for Rett syndrome are fit-for-purpose for clinical trials? June 7, 2023, Nashville, USA

**DOI:** 10.1186/s13063-024-08678-6

**Published:** 2024-12-21

**Authors:** Jenny Downs, Dominique C. Pichard, Walter E. Kaufmann, Joseph P. Horrigan, Melissa Raspa, Gillian Townend, Eric D. Marsh, Helen Leonard, Kathleen Motil, Andrew C. Dietz, Nupur Garg, Amitha Ananth, Breanne Byiers, Sarika Peters, Christopher Beatty, Frank Symons, Aleksandra Jacobs, James Youakim, Bernhard Suter, Paramola Santosh, Jeffrey L. Neul, Tim A. Benke

**Affiliations:** 1 The Kids Research Institute Australia, Centre for Child Health Research, University of Western Australia, 15 Hospital Avenue, Nedlands, Perth, WA 6009 Australia; 2 Curtin School of Allied Health, Curtin University, GPO Box U1987, Perth, WA 6845 Australia; 3 International Rett Syndrome Foundation, 4500 Cooper Road, Suite 204, Cincinnati, OH 45242 USA; 4 Department of Human Genetics, Emory University School of Medicine, 615 Michael St, Atlanta, GA 30322 USA; 5 Department of Neurology, Boston Children’s Hospital, 300 Longwood Ave, Boston, MA 02115 USA; 6 Duke Center for Autism and Brain Development, Duke University, 2608 Erwin Road, Suite 300, Durham, NC 27705 USA; 7 RTI International, 3040 East Cornwallis Road, Research Triangle Park, Durham, NC 27607 USA; 8 School of Psychology and Clinical Language Sciences, University of Reading, Whiteknights Campus, Reading, RG6 6ES UK; 9 Division of Child Neurology and University of Pennsylvania Perelman School of Medicine, Departments of Neurology and Pediatrics, Children’s Hospital of Philadelphia, Philadelphia, PA USA; 10 USDA/ARS Children’s Nutrition Research Center, Baylor College of Medicine, Houston, TX 77030 USA; 11 Shape Therapeutics, Inc, Seattle, USA; 12 University of Alabama at Birmingham, Birmingham, AL USA; 13 Department of Educational Psychology, University of Minnesota, 56 E River Rd, Room 250, Minneapolis, MN 55455 USA; 14 Vanderbilt Kennedy Center, Vanderbilt University Medical Center, 230 Appleton Place, Nashville, TN PMB4037204 USA; 15 Department of Pediatrics, Division of Neurology, Nationwide Children’s Hospital and, The Ohio State University College of Medicine, 700 Children’s Drive, Columbus, OH 43205 USA; 16 Isabelle Rapin Division of Child Neurology, Montefiore Medical Center, Albert Einstein College of Medicine, New York, USA; 17 Acadia Pharmaceuticals Inc., 502 Carnegie Center, Suite 300, Princeton, NJ 08540 USA; 18 Department of Pediatrics & Neurology, Baylor College of Medicine, Houston, TX USA; 19 Department of Child and Adolescent Psychiatry, Developmental Neuropsychiatry & Psychopharmacology, King’s College, London, UK; 20 Centre for Interventional Paediatric Psychopharmacology and Rare Diseases (CIPPRD) & CIPP Rett Centre, Maudsley Hospital, London, UK; 21 HealthTracker Ltd, Gillingham, UK; 22 School of Medicine Depts of Pediatrics, Neurology and Pharmacology, Children’s Hospital Colorado/University of Colorado, 12800 E 19th, MS8102, Aurora, CO 80045 USA

**Keywords:** Rett syndrome, Clinical trial readiness, Clinical outcome assessments, Validation, Meaningful change

## Abstract

**Introduction:**

The clinical, research and advocacy communities for Rett syndrome are striving to achieve clinical trial readiness, including having fit-for-purpose clinical outcome assessments. This study aimed to (1) describe psychometric properties of clinical outcome assessment for Rett syndrome and (2) identify what is needed to ensure that fit-for-purpose clinical outcome assessments are available for clinical trials.

**Methods:**

Clinical outcome assessments for the top 10 priority domains identified in the Voice of the Patient Report for Rett syndrome were compiled and available psychometric data were extracted. The clinical outcome assessments measured clinical severity, functional abilities, comorbidities and quality of life, and electrophysiological biomarkers. An international and multidisciplinary panel of 29 experts with clinical, research, psychometric, biostatistical, industry and lived experience was identified through International Rett Syndrome Foundation networks, to discuss validation of the clinical outcome assessments, gaps and next steps, during a workshop and in a follow-up questionnaire. The identified gaps and limitations were coded using inductive content analysis.

**Results:**

Variable validation profiles across 26 clinical outcome assessments of clinical severity, functional abilities, and comorbidities were discussed. Reliability, validity, and responsiveness profiles were mostly incomplete; there were limited content validation data, particularly parent-informed relevance, comprehensiveness and comprehensibility of items; and no data on meaningful change or cross-cultural validity. The panel identified needs for standardised administration protocols and systematic validation programmes.

**Conclusion:**

A pipeline of collaborative clinical outcome assessment development and validation research in Rett syndrome can now be designed, aiming to have fit-for-purpose measures that can evaluate meaningful change, to serve future clinical trials and clinical practice.

## Background

Rett syndrome (RTT) is a neurodevelopmental disorder caused primarily by pathogenic loss-of-function variants on the X-linked *MECP2*gene [1]. It is a rare disorder that affects approximately 1 in 9000 liveborn females [2]. The incidence of males with loss-of-function *MECP2* variants, who have a variable but more often severe phenotype [3], is unknown. The *MECP2* gene has many roles in central nervous system function [4] and pathogenic variants have pervasive effects on health and functioning. RTT is characterised by regression of hand and communication skills, the development of hand stereotypies and ongoing impacts on hand, communication and gross motor function [5]. Alongside functional impairments, RTT is associated with co-occurring conditions including epilepsy, poor growth and other gastrointestinal problems, autonomic dysfunction, sleep difficulties and scoliosis [6, 5, 7]. Variation in clinical presentation of functional abilities and co-occurring conditions is in part explained by the type of pathogenic variant [8, 9, 6, 10] but there is also individual variability [11].

There have been many clinical trials, most with small sample sizes or methodological limitations that have prevented solid conclusions [12]. Recently, one clinical trial evaluated the effect of sarizotan, a selective 5-HT1A receptor agonist, on episodes of apnea during awake time, with adequate sample size but study endpoints were not met [13]. A set of trials has evaluated the effectiveness of therapeutics that aim to support Brain Derived Neurotropic Factor pathways [14–16]. Fingolimod was found to be safe in six participants but without signs of efficacy [16]. A phase 2 placebo controlled clinical trial of mecasermin (IGF-1) found worsening of several clinical measures and biomarkers although one measure of stereotypic behaviours and another of social communication improved [15], the trial providing a template for outcome measures in future trials. A programme that evaluated a synthetic version of a tripeptide derived from IGF-1, known as trofinetide, concluded recently with a phase 3 clinical trial finding significant between group differences favouring trofinetide over placebo on the two co-primary endpoints, the Rett Syndrome Behaviour Questionnaire (RSBQ) and the Clinical Global Impression–Improvement scale, and the key secondary endpoint (Communication and Symbolic Behavior Scales-Developmental Profile [CSBS-DP ITC]) [14, 17]. Following this, trofinetide was approved by the US Food and Drug Administration (FDA, https://www.fda.gov/drugs/news-events-human-drugs/fda-approves-first-treatment-rett-syndrome).

More clinical trials are ongoing or planned spearheaded by keen interest in the development and evaluation of new precision therapies that target the underlying molecular mechanisms [18]. Notably, two gene therapy programmes for RTT have commenced; Taysha Gene Therapies is conducting gene therapy trials in adults (NCT05606614) and children (NCT06152237) and Neurogene (NCT05898620) is conducting a gene therapy programme is paediatrics.

Research endeavours that are necessary to achieve satisfactory clinical trial readiness for rare genetic disorders such as RTT include understanding the natural history of the disorder, ensuring fit-for-purpose clinical outcome assessments (COA) are available, and harnessing community and advocacy organisation support [19, 12, 20]. The RTT clinical, research and advocacy communities are striving to achieve each of these clinical trial readiness targets [12].

The International Rett Syndrome Foundation (IRSF) and the Rett Syndrome Research Trust co-hosted a virtual Rett Syndrome Externally Led Patient-Focused Drug Development meeting on March 11, 2022, to guide therapeutic development and inform benefit-risk evaluations when evaluating new therapies. The subsequent *Voice of the Patient Report *reported the most important aspects of RTT for new therapeutics to improve, including domains of functioning such as communication and associated conditions such as gastro-intestinal function and sleep [21]. These findings were mirrored in top concerns provided in the NIH sponsored US Natural History study (U54 HD061222 (Percy), NCT02738281) [22]. Fit-for-purpose COAs are needed for each of these domains.

Recent guidance from the FDA is available for the development and use of COAs [23–26]. A few reviews of COAs in RTT are available; including general reviews [12, 27] and reviews of motor function measures [28] and the RSBQ [29]. Accordingly, we held an international workshop to identify and discuss gaps in COAs available to measure parent-reported priorities, attentive to what is important to affected individuals regarding feeling and function. This paper aims to synthesise the workshop activities and discussions and (1) describe available psychometric properties of COAs for RTT for domains considered important in the Voice of the Patient Report and (2) identify what is needed to ensure that available COAs are fit-for-purpose clinical trials in RTT.

## Methods

### Phase 1—pre-meeting

Available outcome measures that had been used in RTT were identified for domains described in the Voice of the Patient Report as priorities for new therapeutics to improve [21]. Priority domains included communication, hand function, gross motor function, gastrointestinal function, movement disorder, sleep, emotional and behavioural function, and child and caregiver quality of life. Validation data for a biomarker (electroencephalogram, EEG) and measures of clinical severity which encompassed some of the top priority domains were also included. Available COA psychometric data for RTT were compiled. An international and multidisciplinary panel of experts (clinical, research, psychometric, biostatistical, industry, and lived experience expertise) were identified through IRSF networks and publications and invited to attend a half-day workshop on COAs hosted at the conclusion of the IRSF Rett Syndrome Scientific Meeting, June 2023 in Nashville, USA. A representative from the FDA was also invited. For preparation, each expert was provided with an agenda and compiled information on the COAs to review prior to the meeting.

### Phase 2—meeting

The workshop meeting was attended by 29 invited experts, the majority from the USA (*n* = 25). Clinicians represented the fields of neurology (*n* = 7), psychology (*n* = 3), psychiatry (*n* = 2), gastroenterology (*n* = 1) and speech and language pathology (*n* = 1). Seven experts were from industry, three represented patient advocacy (IRSF), two represented lived experience and two were researchers. Twenty-five experts attended in person and four attended online. The attendee from the FDA offered non-binding regulatory authority expertise.

**Fig. 1:**
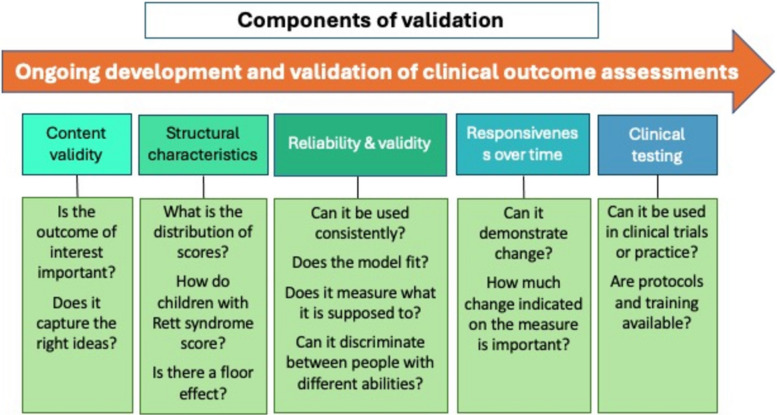
Diagram illustrating the components of clinical outcome assessments necessary for them to be fit-for-purpose

The workshop commenced with background information on the Voice of the Patient Report (DP) and clinical trial readiness in RTT (JN). The concept of fit-for-purpose COAs was defined (JD) (Fig. 1).

This was followed by a series of short presentations on COAs for priority outcomes (Table 1), their strengths and weaknesses, and, if available, data on reliability, validity and responsiveness for RTT. A group discussion on the scope of available COAs for RTT and gaps in validation was then facilitated (TB). Commentary was invited from the FDA representative.

**Table 1:** Summary description of the COAs discussed in the workshop, abbreviations defined in footnotes^a^

Domain		Clinical Outcome Assessments (COAs)	Type of COA^b^	Summary description
Overall global clinical severity		1. Clinical Global Impression – Severity (CGI-S)	ClinRo	Global clinical assessment of severity; 7-point Likert scale with anchors
		2. Clinical Global Impression—Improvement (CGI-I)	ClinRo	Global clinical assessment of change; 7-point Likert scale with anchors
		3. Revised Motor Behavior Assessment (R-MBA)	ClinRo	24 items across 8 domains (Motor dysfunction, Functional skills, Social skills, Aberrant behaviour, Rett behaviours, Seizures, Truncal rocking, Hand stereotypies); scores range from 0 to 96
		4. Global Assessment and Intervention in Rett Syndrome (GAIRS) Checklist	ClinRo	87 items across 10 domains (Basic or pre-requisite behaviour, Neuropsychological abilities, Basic cognitive concepts, Advanced cognitive concepts, Communication abilities, Emotional-affective abilities, Hand motor skills, Graphomotor skills, Global motor abilities, Level of autonomy in daily life); items rated on a 5-point Likert scale
		5. Parent Targeted Visual Analog Scale (PTSVAS) – for parent top 3 concerns	ObsRo	Caregiver rates top 3 of 15 possible treatment concerns on a 0–10 cm visual analogue scale; score is the sum of the three ratings
		6. Rett Syndrome Caregiver Assessment of Symptom Severity (R-CASS)	ObsRo	31 items across four domains (Movement, Communication, Behaviour, RTT-specific symptoms; scores range from 27 to 171
		7. Multi-system Profile of Symptoms Scale (MPSS)	ObsRo	69 items across 12 domains (Mental health problems, Autonomic problems, Cardiac problems, Problems in communication, Problems in social behaviour, Problems in engagement, Gastrointestinal problems, Problems in motor skills, Neurological problems, Orofacial problems, Respiratory problems, Sleep problems); scores range from 0 to 345. Domains scores can also be transformed to 0–5 scores giving a total score of 0–60
Functional abilities assessments	Hand function	1. Rett Syndrome Clinician Rating of Hand Function (RTT-HF)	ClinRo	Global clinical assessment of hand function skills; 7-point Likert scale with anchors
		2. Rett Syndrome Hand Function Scale (RSHFS)	ClinRo	Parents video hand function tasks that demonstrate 1) Grasping objects, 2) Transferring objects and 3) Precision of grasping; hand function rated on a scale of 1 to 8
	Mobility	1. Rett Syndrome Clinician Rating of Ambulation and Gross Motor Skills (RTT-AMB)	ClinRo	Global clinical assessment of standing and walking skills; 7-point Likert scale with anchors
		2. Rett syndrome Gross Motor Scale (RSGMS)	ClinRo	Parents video 15 gross motor tasks that demonstrate (1) Sitting, (2) Standing and walking, (3) Challenge; scores range from 0 to 45
		3. Rett Syndrome Motor Evaluation Scale (RESMES)	ClinRo	Assessment of 25 items that demonstrate (1) Standing, (2) Sitting, (3) Transitions, (4) Walking, (5) Running, (6) Walking up/downstairs; scores range from 0 to 82
		4. Functional Mobility Scale Rett Syndrome (FMS-RS)	ObsRo	Caregivers rate ability to ambulate in therapy (5 m), home (50 m) and community (500 m) settings on a 5-point Likert scale describing the amount of assistance
		5. 2-min walk test adapted to RTT	PerfRo	Distance walked in 2 min, 2 assessors and 1 walk assistant to provided necessary physical support and encouragement required
	Communication	1. Communication and Symbolic Behavior Scales Development Profile Infant Toddler Checklist (CSBS-DP ITC)	ObsRo	24 items across 3 composites [domains] (Social composite, Speech composite, Symbolic composite); scores range from 0 to 57
		2. Rett Syndrome Clinician Rating of Verbal Communication (RTT-VCOM)	ClinRo	Global clinical assessment of verbal communication skills; 7-point Likert scale with anchors
		3. Rett Syndrome Clinician Rating of Ability to Communicate Choices (RTT-COMC)	ClinRo	Global clinical assessment of ability to make choices; 7-point Likert scale with anchors
		4. Observer Reported Communication Ability for Rett syndrome (RTT-ORCA)	ObsRo	80 items across 4 domains (Expressive communication, Receptive communication, Pragmatic communication, Vocabulary for different modalities); one unidimensional score is generated where the average score is 50 and the SD is 10
Co-morbidities	Gastrointestinal	1. Gastrointestinal Health Questionnaire (GHQ)	ObsRo	50 items across 8 domains (1) General health/pain, (2) Eating, chewing, swallowing, (3) Reflux, (4) Gas and bloating, (5) Diarrhoea and constipation, (6) Mood and behaviour, (7) Medication, (8) Surgery); items rated on a 4-point Likert scale
	Movement disorders	1. Clinical monitoring of movement disorders	ClinRo	Observation of freezing, veering, toe walking and hand stereotypies during gait during overground and treadmill walking
		2. Laboratory monitoring of gait	PerfRo	Patterns of lower limb joint kinematics (range and speed of joint movement) during gait on a treadmill
	Sleep	1. Children’s Sleep Habits Questionnaire (CSHQ)	ObsRo	33 items across 8 domains (1) Bedtime resistance, (2) Sleep onset delay, (3) Sleep duration, (4) Sleep anxiety, (5) Night wakings, (6) Parasomnias, (7) Sleep-disordered breathing, (8) Daytime sleepiness); scores range from 33 to 99, score above 41 indicate sleep problems
		2: Sleep Disturbance Scale for Children (SDSC)	ObsRo	26 items across 6 domains (1) Disorders of maintaining sleep, (2) Sleep breathing disorders, (3) Disorders of arousal and nightmares, (4) Sleep wake transition disorders, (5) Disorders of excessive somnolence, (6) Sleep hyperhidrosis); scores range from 26 to 130 and are converted to T scores
		3. Polysomnography (PSG)	Biomarker	Overnight laboratory monitoring of sleep stages and cycles
		4.Wearables (Actigraph and watch for measurement of heart rate)	Biomarker	Monitoring of movement and heart rate with wearables
	Emotional and Behavioural	1. Rett Syndrome Behaviour Questionnaire (RSBQ)	ObsRo	45 items across 8 domains (other items do not load to a domain but are included in the total score): (1) General Mood, (2) Breathing Problems, (3) Hand Behaviours, (4) Repetitive Face Movements, (5) Body rocking and expressionless face, (6) Night-time Behaviours, (9) Fear/Anxiety and (8) Walking/Standing (2 items); 3-point Likert scale, scores range from 0 to 90
		2. Aberrant Behavior Checklist—Community Edition (ABC-C)	ObsRo	58 items across 5 domains/independent subscales (1) Irritability, (2) Social withdrawal/lethargy, (3) Hyperactivity, (4) Stereotypy, (5) Inappropriate Speech; scores range from 0 to 174
		3. Anxiety, Depression, and Mood Scale (ADAMS)	ObsRO	28 items across 5 domains ((1) Manic/hyperactive behaviour, (2) Depressed mood, (3) General anxiety, (4) Social avoidance, (5) Obsessive compulsive behaviour); items rating on a 4-point Likert scale, domain scores and not total scores are calculated
Electrophysiological biomarkers		1. Visual Evoked Potentials (VEP)	Biomarker	Measure of the electrical signal generated at the visual cortex in response to visual stimulation
		2. Auditory Evoked Potentials (AEP)		Measure of the electrical signal generated in the brain in response to visual stimulation
		3. Resting state EEG		Measure the electrical signal in the brain in a relaxed non stimulated individual
Quality of life		1. Child Health Questionnaire-50 (CHQ-50)	ObsRO	50 items in 2 subscales (Physical and Psychosocial summary scores), each scaled from 1 to 100
		2. Quality of Life Inventory – Disability (QI-Disability)	ObsRO	32-item scale across 6 domains ((1) Physical health, (2) Positive emotions, (3) Negative emotions, (4) Social interactions, (5) Leisure and the outdoors, (6) Independence); domain and total scores range from 0 to 100
		3. Rett Syndrome Caregiver Burden Inventory (RTT-CBI)	PRO	26 items across 4 domains ((1) Physical burden, (2) Emotional burden, (3) Social burden, (4) Time dependence); total scores range from 26 to 130

^a^*R-MBA* Motor Behavior Assessment – Revised, *R-CASS* Rett Caregiver Assessment of Symptom Severity, *MPSS* Multi-system Profile of Symptoms Scale, *CSBS-DP ITC* Communication and Symbolic Behavior Scales-Developmental Profile Infant Toddler Checklist, *RTT-VCOM* Rett Syndrome Clinician Rating of Verbal Communication, *RTT-COMC* Rett Syndrome Clinician Rating of Ability to Communicate Choices, *RTT-ORCA* Observer Reported Communication Ability for Rett syndrome, *RTT-HF* Rett Syndrome Clinician Rating of Hand Function, *RSHFS* Rett Syndrome Hand Function Scale, *RTT-AMB* Rett Syndrome Clinician Rating of Ambulation and Gross Motor Skills, *RSGMS* Rett syndrome Gross Motor Scale, *RESMES* Rett Syndrome Motor Evaluation Scale, *FMS-RS* Functional Mobility Scale Rett Syndrome, *GHQ* Gastrointestinal Health Questionnaire, *CSHQ* Children’s Sleep Habits Questionnaire, *SDSC* Sleep Disturbance Scale for Children, *PSG* Polysomnography, *RSBQ* Rett Syndrome Behaviour Questionnaire, *ABC-C* Aberrant Behavior Checklist—Community Edition, *ADAMS* Anxiety, Depression, and Mood Scale, *VEP* Visual Evoked Potentials, *AEP* Auditory Evoked Potentials, *CHQ* Child Health Questionnaire, *QI-Disability* Quality of Life Inventory – Disability, *RTT CBI* Rett Syndrome Caregiver Burden Inventory

^b^*ClinRo* Clinician reported outcome, *ObsRo* Observer reported outcome, *PRO* Patient reported outcome, *PerfO* Performance outcome measure

### Phase 3—post-meeting

A survey was administered on REDCap™ to supplement the workshop discussion and collect further feedback on gaps in validation, by each of the COA domains discussed. The domain of quality of life was reviewed in the survey because of limited time during the workshop. Open-ended questions sought feedback on use of total or domain scores, meaningful change, how COAs should be implemented in clinical trials and new directions that the RTT research community should be taking. Feedback was collated in an Excel spreadsheet and coded (JD) using inductive content analysis [30].

The COnsensus-based Standards for the selection of health status Measurement INstruments (COSMIN) checklist was used to evaluate the methodological quality of each parent-reported COA [31]. The COSMIN checklist rates content validity, structural validity, internal consistency, cross-cultural validity/measurement invariance, reliability, measurement error, construct validity and responsiveness. Each property for each patient-reported outcome measure was rated as “doubtful”, “inadequate”, “adequate”, or “very good” for quality following COSMIN Risk of Bias criteria [32].

## Results

### COA validation

Overall, internal consistency data and known group comparisons for construct validity had been evaluated for most instruments. Structural validity and reliability had been assessed for fewer scales but for the Multi-system Profile of Symptoms Scale (MPSS) [33], Observer Reported Communication Ability for Rett syndrome (RTT-ORCA) [34], RSBQ [35], Quality of Life Inventory – Disability (QI-Disability) [36] and the Rett Syndrome Caregiver Burden Inventory (RTT-CBI) [37]. There was limited evidence to support content validity, in particular parent-informed relevance, and no data on meaningful change or cross-cultural validation. Completed clinical trials have provided evidence of responsiveness for a few COAs. Table 2 presents the quality of available validation data for the parent-reported measures.

**Table 2:** Methodological quality of psychometric evaluations for the parent-reported measures, rated using the COnsensus-based Standards for the selection of health status Measurement INstruments (COSMIN) Risk of Bias criteria [32]. Clinical outcome assessments are defined in the footnote^a^

Domain	Clinical Outcome Assessment	Author, year	Content validity		Internal structure			Reliability	Measurement Error	Hypotheses testing for construct validity	Responsiveness^c^
			**Development**^**b**^	**Content Validity**	**Structural validity**	**Internal Consistency**	**Cross-Cultural Validity/ Measurement Invariance**				
Global Severity	Parent Targeted Visual Analog Scale (PTSVAS) – for parent top 3 concerns	O’Leary et al. 2018 [15] PMID: 29,560,377	-	-	-	-	-	-	-	9b Doubtful	-
	MPSS	Singh et al. 2022 [33] PMID: 36,079,020 Leoncini et al. 2024 PMID: 38,952,469	1a Adequate	1b Adequate	3 Adequate	4 Very good	-	6 Very good	-	9a Adequate	-
	R-CASS	Raspa et al. 2023 [44] PMID: 38,438,817	-	-	3 Adequate	4 Very good	-	-	-	9a Very good 9b Very good	-
Communication	CSBS-DP ITC	O’Leary et al. 2018 [15] PMID: 29,560,377 Neul et al. 2024 [17] PMID: 38,232,652	Existing tool	-	-	-	-	-	-	-	Social score 10d Very good
	RTT-ORCA	Reeve et al. 2023 [34] PMID: 37,536,121	1a Very good	1b Very good 2a Very good	3 Adequate	4 Very good	-	6 Very good	-	9a Adequate 9b Very good	-
Mobility	FMS-RS	Stahlhut et al. 2017 [51] PMID: 27,558,323	Modification of existing tool	-	-	-	-	6 Very good	-	9a Very good 9b Very good	-
Gastrointestinal	GHQ	Motil et al. 2021 [60] PMID: 32,969,958	1 Adequate	2 Adequate	-	4 Very good	-	-	-	9b Very good	-
Sleep	SDSC	Boban et al. 2016 [61] PMID: 27,255,190	Existing tool	-	-	-	-	-	-	9b Very good	-
	CSHQ	Veatch et al. 2021 [62] PMID: 34,388,423	Existing tool	-	-	-	-	-	-	9b Doubtful	-
Emotional Behavioural	RSBQ	Mount et al. 2002 [66] PMID: 12,455,930 Mount et al. 2003 [67] PMID: 12,475,362 Barnes et al. 2015 [65] PMID: 26,379,794 Oberman et al. 2023 [35] PMID: 37,104,862 Percy et al. 2023 [29] PMID: 37,635,789 Downs et al. 2024 [68] PMID: 39,455,915	1 Adequate	2 Doubtful	3 Very good	4 Very good	-	6 Doubtful	-	9a Very good 9b Very good	Total Score 10d Adequate
	ABC-C	Barnes et al. 2015 [65] PMID: 26,379,794 O’Leary et al. 2018 [15] PMID: 29,560,377	Existing tool	-	-	4 Very good	-	-	-	9a Very good 9b Very good	ABC-Stereotypy Subscale—10d Doubtful
	ADAMS	Khwaja et al. 2014 [64] PMID: 24,623,853 Barnes et al. 2015 [15] PMID: 26,379,794 O’Leary et al. 2018 [15] PMID: 29,560,377	Existing tool	-	-	4 Very good	-	6 Very good	-	9a Very good 9b Very good	Social Avoidance Domain - 10d Doubtful Depressed Mood Domain – 10d Very good
Quality of life	CHQ	Lane et al. 2011 [79] PMID: 22,013,176	Existing tool	-	-	4 Very good	-	-	-	9b Very good	10c Very good (based on change with time)
	QI-Disability	Downs et al. 2019 [36] PMID: 30,460,513 Epstein et al. 2019 [75] PMID: 31,163,096 Jacoby et al. 2020 [74] PMID: 32,412,990 Williams et al. 2021 [78] PMID: 32,862,445 Reddihough et al. 2021 [77] PMID: 33,885,172 Mendoza et al. 2021 [76] PMID: 32,843,489	1 Very good	2a Very good 2b Very good 2c Very good	3 Adequate	4 Very good	-	6 Very good	Very good	9b Very good	10b Very good
	RTT-CBI or the Rett syndrome Caregiver Inventory Assessment (RTT CIA)	Lane et al. 2017 [37] PMID: 28,132,121	Modified existing tool 1 Doubtful	2 Doubtful	3 Very good	4 Very good	-	6 Doubtful	-	9b Very good	-

^a^*MPSS* Multi-system Profile of Symptoms Scale, *R-CASS* Rett Caregiver Assessment of Symptom Severity, *CSBS-DP ITC* Communication and Symbolic Behavior Scales-Developmental Profile Infant Toddler Checklist, *RTT-ORCA* Observer Reported Communication Ability for Rett syndrome, *FMS-RS* Functional Mobility Scale Rett Syndrome, *GHQ* Gastrointestinal Health Questionnaire, *SDSC* Sleep Disturbance Scale for Children, *CSHQ* Children’s Sleep Habits Questionnaire, *RSBQ* Rett Syndrome Behaviour Questionnaire, *ABC-C* Aberrant Behavior Checklist—Community Edition, *ADAMS* Anxiety, Depression, and Mood Scale, *CHQ* Child Health Questionnaire, *QI-Disability* Quality of Life Inventory – Disability, *RTT-CBI* Rett Syndrome Caregiver Burden Inventory

^b^Reviewed if developed or validated for a RTT population

^c^Evidence enabled rating of responsiveness to change for 3 of 4 categories of the COSMIN Risk of Bias criteria—Construct approach (i.e. hypotheses testing; comparison with other outcome measurement instruments); 10c. Construct approach: (i.e. hypotheses testing: comparison between subgroups); 10d. Construct approach: (i.e. hypotheses testing: before and after intervention). Each rating is preliminary because it is based on small number of studies and replication is needed

#### Overall global severity

##### Clinician-reported

The RTT Clinician Global Impression Scales are rated for patient severity (CGI-S) or improvement (CGI-I) using anchor descriptors on a 7-point Likert scale [38]. In preparation for the trofinetide trials, site clinicians participated in periodic training where they each rated vignettes to obtain uniform alignment of assessments based on an expert rater; differences in ratings of 1 point were considered reliable [38]. Familiarity with RTT and the descriptors are central to the use of the CGI-S and CGI-I [38]. Validity data are not available although evidence of responsiveness was suggested in the paediatric Phase 2 [39] and 3 [14] trofinetide trials.

Multi-domain clinician-reported severity assessments include the Revised Motor-Behavior Assessment (R-MBA) [40], modified from the Motor-Behavior Assessment [41], and the Global Assessment and Intervention in Rett Syndrome (GAIRS) Checklist [42]. Using data for more than 1000 individuals with RTT, the R-MBA comprises a five-factor model for motor dysfunction, functional skills, social skills, aberrant behaviour and respiratory behaviours [40]. Psychometric evidence indicated good model fit, internal consistency and hypothesis testing for construct validity [40]. The GAIRS checklist focuses on 85 skills that were grouped into 10 subscales describing a range of functional abilities and adaptive behaviours [42]. Although factor analysis suggested two factors, analysis of the original groupings of items suggested good internal consistency, test retest reliability and expected relationships with other measures [42].

##### Caregiver-reported

The MPSS is an electronic caregiver-report tool comprising 12 individually validated sub-scales that measure mental health, autonomic, cardiac, communication, social behaviour, engagement, gastrointestinal, motor skills, neurological, orofacial, respiratory and sleep domains, which explore symptom frequency in the past month, supplemented with additional 5 subscales that describe sensory, immune and infection, endocrine, skeletal and dermatological domains for the evaluation of changes over a longer period [33]. Items were developed from the literature with parent and clinician feedback giving adequate evidence for content validity. There was evidence for adequate structural validity and construct validity, very good internal consistency [33] and scores correlated with physiological monitoring in 10 children and adults with RTT [43].

Newly published, the Rett Caregiver Assessment of Symptom Severity (R-CASS) [44] is a 34-item measure comprising Movement, Communication, Behaviour and RTT-specific domains. The four-factor model showed good fit statistics, very good internal consistency, expected relationships with related measures and differences between the different age and variant groups, suggesting very good construct validity [44].

#### Functional abilities

##### Clinician-reported

Single-item rating scales were recently developed for RTT-specific domains, including verbal communication (RTT-VOM), communication of choices (RTT-COMC), ambulation (RTT-AMB) and hand use (RTT-HF). Scoring anchors were developed by literature review, clinical experience and iterative review by the investigators suggesting some content validity [45]. The RTT-COMC showed responsiveness in the phase 3 trofinetide trial [14, 17]; however, no other validation data are yet available.

Two measures evaluate gross motor and hand function abilities through collection of video data. Parents are provided with checklists that guided them for filming video clips of motor function at home, which are subsequently uploaded to a protected server [46, 47]. The Rett Syndrome Hand Function Scale (RSHFS) evaluates grasping, holding and transferring a range of large and small objects [46]. Scores capture a range of functioning [46] with evidence supporting construct validity [46], and longitudinal analysis after 3 to 4 years suggesting that approximately one third of children with RTT lose hand function skills, particularly the younger ones [47]. Following training, inter- and intra-rater reliability are good [48]. The Rett Syndrome Gross Motor Scale (RSGMS) evaluates sitting, standing, transfer and walking tasks, with evidence that it captures a range of functioning, has good factor structure, internal consistency, construct validity and test–retest reliability [49]. The Rett Syndrome Motor Evaluation Scale (RSMES) is administered by a therapist and includes additional transition and walking on stairs items to those in the RSGMS and there is similarly good evidence for model fit, internal consistency, construct validity and inter-rater reliability [50].

One performance measure for walking was identified. The 2-min walk test (2MWT) was modified from the 6-min walk, a submaximal exercise test used to assess aerobic capacity and endurance [51]. Strategies have been designed to enhance how the individual with RTT understands the task and can be motivated to perform their best effort [51]. Correlations between the 2MWT scores, clinical severity and gross motor skills were as hypothesised, test–retest reliability was good, and the minimal detectable difference was 38 m [51]. Clinical and laboratory assessment of gait were discussed noting that specification of the outcome and validation data are lacking [52, 53].

The Vineland Adaptive Behavior Scales (VABS) and the Mullen Scales of Early Learning (MSEL) were not discussed at the meeting but have been used in clinical trials for RTT. The VABS is a widely used scale of adaptive behaviours [54]. For RTT, there is evidence of a floor effect [55]. It had limited use in the dextromorphan [56] and Phase 2 IGF-1 [15] clinical trials while it is included in the protocol of the current adult Taysha gene therapy trial (NCT05606614). The MSEL is also a widely used developmental assessment which was modified for RTT to enable individuals to take longer to for tasks and use eye gaze for communication, because impaired motor and expressive language skills confound the evaluation of developmental domains [57]. Individuals with RTT scored higher on the visual reception and receptive communication domains when using the modified scale than the original scale, and there were good correlations between modified MSEL and VABS scores [57]. It was used in the IGF-1 phase 2 trial [15], while the original MSEL was implemented in the dextromorphan study [56]. The available modifications to the MSEL are a positive step and adaptations to other assessments of development and adaptive behaviours may also be necessary.

##### Caregiver-reported

The CSBS-DP ITC is an autism screening tool developed for children younger than 2 years [58]. Validation data for RTT are lacking but significant between group effects were found in the Phase 2 IGF-1 [15] and Phase 3 trofinetide [17, 14] trials suggesting some responsiveness. Initially developed for Angelman syndrome [59], the ORCA was modified for RTT, and evidence supports its content validity, reliability and construct validity [34].

#### Single co-morbidities

##### Caregiver-reported

Caregiver-reported measures for gastrointestinal health, sleep and emotions and behaviour were discussed (Table 2). Data to support content validity, internal consistency and construct validity are reported for the Gastrointestinal Health Questionnaire (GHQ) [60]. Validation data for parent-report sleep questionnaires are particularly sparse [61, 62]. In a small pilot study (*n*= 7, 4 to 16 years), accelerometer and physiological data was able to classify sleep stages with 85.1% accuracy compared with polysomnography [63]. The RSBQ, Aberrant Behavior Checklist—Community Edition (ABC-C) and Anxiety, Depression, and Mood Scale (ADAMS) have been used to measure emotions and behaviours in clinical trials [64, 14, 15]. There are data to support internal consistency and construct validity in RTT for the ABC-C [65] and ADAMS [65]. Responsiveness in the Phase 2 IGF-1 trial was demonstrated for ABC-C Stereotypy but not for other ABC-C subscales or the ADAMS [15]. The RSBQ was initially developed to identify behavioural features of RTT [66, 67] rather than measure change, but it has been used in multiple clinical trials. There is some evidence for content validity, internal consistency, factor structure and known groups hypothesis testing [66, 35, 68], and test–retest reliability may be poor [69]. The RSBQ was one of two primary endpoints demonstrating significant between group change [14] in the Phase 3 trofinetide trial, following demonstration of efficacy in the preceding paediatric Phase 2 study [39].

##### Biomarker—EEG

A responsive biomarker has not been demonstrated in any interventional clinical trials in RTT. The use of quantitative EEG and evoked potentials as a biomarker of severity in observational studies was reviewed, with some evidence of construct validity [70–73].

#### Quality of life

##### Caregiver-reported

Two measures of child quality of life have validation data for RTT. QI-Disability is a 32-item questionnaire with evidence that supports its content validity, reliability and responsiveness to altered health status [36, 74–78]. The Child Health Questionnaire-50 is a 50-item questionnaire with evidence to support internal consistency and construct validity, with some data demonstrating responsiveness over long periods of time [79]. The RTT-CBI, a questionnaire measuring the impact on caregivers of having a child with RTT, has good evidence for structural validity, internal consistency and construct validity (Table 2) [37].

### Evaluation of needs

The meeting discussion and survey focused on identifying gaps and limitations of the reviewed measures related to their (1) administration, (2) development of COA content and (3) psychometric properties. A fourth category considered the value of domain and total scores as trial outcomes (Table 3).

**Table 3:** Categories and elements of post-meeting feedback on the gaps and limitations, classified by domains or groups of COAs. Acronyms for each measure are presented in a footnote^a^

Domain	Measures	Categories	Elements of gaps and limitations
Overall global clinical severity	Clinician Global Impression Scales	Administration	• Ensure capacity for consistent rating by clinicians and parents through training • Ensure capacity for ongoing training during trials to prevent drift
		Measure content	• Consider development of new anchors for symptoms such as behaviour change • Publish a grid version of the CGI-I to accompany the grid version of the CGI-S • Consider refinements of anchors
		Psychometric properties	• Identify meaningful change
	Multi-item severity scales 1. R-MBA 2. R-CASS	Administration	• Ensure capacity for standardised administration
		Psychometric properties	• Complete reliability, validation and responsiveness testing • Compare and contrast with the RSBQ, CGI-S and CGI-I
	MPSS	Administration	• Generate a standard protocol and administrator training/education around its use
		Measure content	• Value to have focus on range of symptoms including autonomic symptoms
		Psychometric properties	• Profiles for different age, variant and severity groups • Complete responsiveness testing • Map sensitivity to change against other clinical outcomes
Functional abilities assessments	Communication 1. CSBS-DP ITC 2. RTT-VCOM 3. RTT-COMC 4. RTT-ORCA	Administration	• Standardised administration protocols are needed • Ensure capacity to use AAC devices is incorporated into communication measures
		Measure content	• Validate the communication measures using direct assessment • Consider more use of eye tracking for evaluation of receptive communication • Videotape parents describing communication vignettes to capture change
		Psychometric properties	• Profiles for different age, variant and severity groups for each measure • Some validation data available for the CSBS-DP ITC and the RTT-ORCA but complete reliability, validation and responsiveness testing needed • Examine correlations between the communication measures and identify when they are each most appropriate to use • Consolidate validation efforts on the most appropriate measure/s • Identify meaningful change
	Hand function 1. RTT-HF 2. RSHFS	Administration	• Consider the advantages of video assessment in the RSHFS and central rating, potentially as a biomarker • Need to ensure can administer across trial sites
		Psychometric properties	• Examine correlations between hand function measure data and other COA data • Identify meaningful change
	Mobility 1. RTT-AMB 2. RS-GMS 3. RESMES 4. FMS-RS 5. 2-min walk test adapted to RTT	Psychometric properties	• Needing multicentre collaboration to generate larger datasets for characterisation and validation • Comprehensive validation data needed for most of these measures • Consider modification of the 10 m walk test to be able to capture and measure walking abilities in a larger proportion of the RTT population
Co-morbidities and associated clinical issues	Gastrointestinal 1. GHQ	Psychometric properties	• Some validation data available on content and convergent validity but needs additional reliability, validation and responsiveness testing • Identify meaningful change
	Movement disorders 1. Clinical monitoring 2. Laboratory gait assessment	Administration	• Costly to collect gait laboratory data and still at an exploratory stage • Multiple sites will need to collaborate to generate these data but processes to do this are unclear
		Psychometric properties	• Comprehensive validation data needed • Consider wearables for analysis of movement disorders (e.g. as in Parkinson’s disease) and validate against video measures • No valid measure of hand stereotypies
	Sleep 1. CSHQ/SDSC 2. PSG sleep studies 3. Wearables (Actigraph and watch)	Administration	• Need to ensure multisite data can be collected for PSG studies
		Psychometric properties	• Uncertain relationships between PSQ and other sleep measure data • Consider other parent-report questionnaires that may have fewer non-applicable items for children with disability—e.g. the SNAKE and the Sleep Disturbance Questionnaire for children • Consider use of wearables in sleep as in Parkinson’s disease • Comprehensive questionnaire validation data needed in RTT, including updates on ongoing wearables projects in RTT needed (e.g. Emerald measure of sleep)
	Emotional and Behavioural 1. RSBQ 2. ABC-C 3. ADAMS	Administration	• Open-source training videos for parents (standardisation)
		Psychometric properties	• Establish score profiles for different age, variant and severity groups • Patchy validation data in RTT available for most of these measures, even for the RSBQ, which needs to be expanded to include reliability, additional components of validity, and responsiveness testing • Expand longitudinal datasets to examine trajectories
Electrophysiological biomarkers	1. VEP 2. AEP	Administration	• Standardised protocols and stimulus protocols • How to increase the proportion of useable data
		Psychometric properties	• Need to collect cross-sectional and longitudinal data at more sites, to accumulate larger sample sizes and profiles for different age, variant and severity groups • Test–retest reliability • Relationships between EEG data and clinical outcomes, including their meaningful change • Change in response to medications
Quality of Life*	Child QOL 1. CHQ 2. QI-Disability Parent QOL 1. RTT CBI	Psychometric properties	• Need to compare both child QOL measures, test responsiveness of the CHQ • More validation data needed on the RTT CBI including responsiveness

*R-MBA* Motor Behavior Assessment – Revised, *R-CASS* Rett Caregiver Assessment of Symptom Severity, *MPSS* Multi-system Profile of Symptoms Scale, *CSBS-DP ITC* Communication and Symbolic Behavior Scales-Developmental Profile Infant Toddler Checklist, *RTT-VCOM* Rett Syndrome Clinician Rating of Verbal Communication, *RTT-COMC* Rett Syndrome Clinician Rating of Ability to Communicate Choices, *RTT-ORCA* Observer Reported Communication Ability for Rett syndrome, *RTT-HF* Rett Syndrome Clinician Rating of Hand Function, *RSHFS* Rett Syndrome Hand Function Scale, *RTT-AMB* Rett Syndrome Clinician Rating of Ambulation and Gross Motor Skills, *RSGMS* Rett syndrome Gross Motor Scale, *RESMES* Rett Syndrome Motor Evaluation Scale, *FMS-RS* Functional Mobility Scale Rett Syndrome, *GHQ* Gastrointestinal Health Questionnaire, *CSHQ* Children’s Sleep Habits Questionnaire, *SDSC* Sleep Disturbance Scale for Children, *PSG* Polysomnography, *RSBQ* Rett Syndrome Behaviour Questionnaire, *ABC-C* Aberrant Behavior Checklist—Community Edition, *ADAMS* Anxiety, Depression, and Mood Scale, *VEP* Visual Evoked Potentials, *AEP* Auditory Evoked Potentials, *CHQ* Child Health Questionnaire, *QI-Disability* Quality of Life Inventory – Disability, *RTT CBI* Rett Syndrome Caregiver Burden Inventory

^a^This domain was not discussed in the meeting because of time restraints and participants were directed to pre-meeting materials

#### Category 1: Administration of measures

The meeting participants identified needs for standardised administration protocols across measures to achieve consistency among sites, reliable rating and coding, and for maintaining consistency along the course of a clinical trial. Multisite collaborations were needed to implement the measures regularly in clinical practice, to enable common understandings of the content, how to administer the measures, and build cross-sectional and longitudinal datasets for validation analyses. Specific feedback described the need for EEG protocol development to increase the proportion of useable data consistently across sites and for modification of available communication measures to ensure that capacity to use Augmentative and Alternative Communication devices to communicate language is recognised and scored. There was support for ongoing research evaluating home-based assessments such as the RSHFS [46] and the RSGMS [49], because of the ability to capture these motor functions in a natural environment as well as the potential to reduce family burden of travel to trial sites while using an independent central rater.

#### Category 2: COA content

There was very little parent-informed content validation for the measures, essential for both clinician and parent-reported measures, to ensure that items are relevant and comprehensively describe the domain of concern, and comprehensible to the respondent [32]. RTT-ORCA and QI-Disability are supported by very good evidence for their content validity, possibly reflecting more recent development in line with FDA recommendations [80]. In alignment with more widespread use of augmentative and alternative communication in RTT [81], further development of direct assessments using eye tracking for evaluation of receptive communication skills was discussed, building on the modifications for RTT of the MSEL [57].

#### Category 3: Psychometric properties

The panel recognised multiple needs for more comprehensive reliability, validity and responsiveness data across the COAs. Several COAs were available for some domains but the relationships of scores between each measure were not known nor why one measure would be chosen over another for a particular group of children or purpose. Establishing score profiles for different age, variant and severity groups and metrics for meaningful change were noted by the panel for some COAs, but arguably these needs apply to all COAs.

Data to support interpretation of the meaning of score changes was particularly sparse. The minimal detectable difference (MDD) for the Rett Syndrome Gross Motor Scale has been published [49] but no other MDD values are available for any other measures, limiting evaluation of whether change in scores is due to underlying measurement variability or actual change. Across all measures, no information was available to describe minimal clinically important change values for groups or change that is meaningful for individuals [82]. The participants acknowledged that evidence of change will be generated within clinical trials. With this in mind, in the FDA approval of trofinetide for the treatment of RTT, statistically significant changes of 0.5 (versus 0.2 for placebo) in the CGI-I was associated a change of 4.9 (versus 1.7 for placebo) in the RSBQ [14]. While this is not a measure of MDD, it represents the current benchmark of responsiveness. Exit interviews in clinical trials have a critical role in explaining the meaning of any between group differences [83]. A novel qualitative approach for videotaping parents describing communication vignettes to capture change in skills was suggested.

#### Category 4: Value of total and domain scores

The panel recognised that both total and domain scores could have utility, depending on the measure and context. The rationale in favour of prioritising domain scores considered the direct relevance of the domain to the priority outcome and potential for change within a specific domain to be observed before changes in an overall score. Total scores were considered of value if strong validation evidence and/or reference values were available. Composite approaches that describe multiple domains can represent multiple co-existing functional deficits and comorbidities. It was noted that FDA input in this regard remains deferential to the specifics of the individual trial protocols.

## Discussion

Steered by the IRSF, this workshop was organised in response to the growing number of clinical trials for RTT, including two gene therapy programmes. As has been done in other rare disease groups, (e.g. [84, 19]) we harnessed the expertise of an international, multidisciplinary, and cross-sectoral group of experts to critically appraise whether COAs used in RTT, for the top 10 priorities identified in the *Voice of the Patient Report*, are fit-for-purpose. We suggest that future research should be directed to filling the identified validation gaps, with oversight from the RTT advocacy organisations, whose goals include ensuring clinical trial readiness. We also note the commitment of the Rett Syndrome Research Trust to outcome measures research and their recent initiatives for parent-report [34] and biomarker [85] measures (https://reverserett.org/research/initiatives/clinical-initiatives/).

In the short term, some needs are simpler to address. For example, administration protocols and training materials need to be developed, including for remote assessments to reduce patient travel to study sites and support inclusive enrolment and participant retention in trials. Content validity needs to be evaluated by engaging with clinicians and parent end-users using qualitative methods, followed by pilot testing if modifications are made. Examining the relationships between measures using available datasets, such as the US Natural History Study, are recommended. We note that new disease-changing treatments could “unmask” problematic behaviours and emotions because of improved motor or communication skills and that, therefore, ongoing validation of behaviour measures is recommended.

More complex research studies are needed to analyse temporal psychometric properties. Evaluation of test–retest reliability over a short period of time (e.g. 1 month) would inform reliability and the minimal detectable difference, if anchor questions are also used (e.g. [74]) If also accompanied by questions about change in the domain, anchor-based methodologies could be used to estimate values for minimal clinically important differences [86]. Longer-term evaluations, e.g. 3 monthly assessments over a 12-month period, would inform short-term trajectories and stability over the duration of most clinical trials. A comprehensive programme of research needs to be designed to establish how to measure sleep, including evaluations of parent-report sleep questionnaires [87, 88] and wearables [89]. Remote assessment of physiological indicators could be sensitive to change in response to new treatments, even preceding changes in observable functional skills and behaviours, while offering a window into more typical functioning within the home/natural environment. Ongoing validation of EEG biomarkers is needed for them to be fit-for-purpose.

Longer-term goals could include establishing a RTT Toolbox. This would comprise a set of well-validated measures and standardised protocols, including vignettes for evaluation of global severity and domains such as functional abilities, and guides for collecting EEG biomarkers. The Toolbox could also include access to baseline datasets collected in clinical trials, to complement the natural history data available to date. The development of a RTT Toolbox would be best served by a multisite, multidisciplinary and international collaboration of consumers, researchers and clinicians. Critical to these efforts is the inclusion of industry collaborators in the development and refinement of tools, aiming at a more direct and regulatory-focused interaction that includes sharing of clinical trial baseline data.

## Conclusions

This workshop and its subsequent evaluation have identified necessary research activities with potential to yield benefits, not only for the evaluation of new therapeutics in clinical trials but also for monitoring clinical care. With direct participation of the international RTT community, efforts aimed at improving and expanding currently available outcome measures will be planned and made to address priority domains identified in the Voice of the Patient Report to be fit-for-purpose, working towards a vision of establishing a RTT Toolbox. Because the community of RTT stakeholders is small, and resources are limited, a well-designed international research collaboration is essential for success.

## Data Availability

Not applicable. All data are available in the literature or in the manuscript.

## Abbreviations

RTT: Rett syndrome
RSBQ: Rett Syndrome Behaviour Questionnaire
CGI-I: Clinical Global Impression–Improvement
CGI-S: Clinical Global Impression–Severity
CSBS-DP ITC: Communication and Symbolic Behavior Scales-Developmental Profile Infant Toddler Checklist
COA: Clinical Outcome Assessments
IRSF: International Rett Syndrome Foundation
FDA: Food and Drug Administration
EEG: Electroencephalogram
COSMIN: COnsensus-based Standards for the selection of health status Measurement Instruments
R-MBA: Motor Behavior Assessment – Revised
R-CASS: Rett Caregiver Assessment of Symptom Severity
MPSS: Multi-system Profile of Symptoms Scale
RTT-VCOM: Rett Syndrome Clinician Rating of Verbal Communication
RTT-COMC: Rett Syndrome Clinician Rating of Ability to Communicate Choices
RTT-ORCA: Observer Reported Communication Ability for Rett syndrome
RTT-HF: Rett Syndrome Clinician Rating of Hand Function
RSHFS: Rett Syndrome Hand Function Scale
RTT-AMB: Rett Syndrome Clinician Rating of Ambulation and Gross Motor Skills
RSGMS: Rett Syndrome Gross Motor Scale
RESMES: Rett Syndrome Motor Evaluation Scale
FMS-RS: Functional Mobility Scale Rett Syndrome
GHQ: Gastrointestinal Health Questionnaire
CSHQ: Children’s Sleep Habits Questionnaire
SDSC: Sleep Disturbance Scale for Children
PSG: Polysomnography
ABC-C: Aberrant Behavior Checklist—Community Edition
ADAMS: Anxiety, Depression, and Mood Scale
VEP: Visual evoked potentials
AEP: Auditory evoked potentials
CHQ: Child Health Questionnaire
QI-Disability: Quality of Life Inventory – Disability
RTT CBI: Rett Syndrome Caregiver Burden Inventory
VABS: Vineland Adaptive Behavior Scales
MSEL: Mullen Scales of Early Learning
